# Supplementary material for: Direct SARS-CoV-2 infection of the human inner ear may underlie COVID-19-associated audiovestibular dysfunction
Source: Commun Med (Lond). 2021 Oct 29;1:44. doi: 10.1038/s43856-021-00044-w (PMC8633908; doi:10.1038/s43856-021-00044-w)
Supplement: Supplementary file 1 — Supplementary information. [file 43856_2021_44_MOESM1_ESM.pdf]

Supplementary Information: Direct SARS-CoV-2 infection of the human inner ear may underlie COVID-19-associated audiovestibular dysfunction

Supplementary Note 1: Patient summaries

Patient #1

A 55-year-old, previously healthy Hispanic podiatrist with no prior otologic history and no active medical problems presented with isolated, sudden right sided hearing loss. No associated dyspnea, anosmia, fever or chills. Physical examination revealed normal appearing ear canals and tympanic membranes with aerated middle ears bilaterally. Audiometric testing revealed right-sided sensorineural hearing loss (Fig. 1a). He was started on a 60mg prednisone burst with anticipated taper after 14 days. Subsequent MRI of the brain revealed no retrocochlear pathology to explain his hearing loss. A day after starting on prednisone, he received intratympanic dexamethasone (10 mg/ml). Partial response to the glucocorticoid treatment was recorded during a 7-day follow up. The following day he developed dyspnea and fever of 102°F. COVID-19 testing was ordered (based on qRT-PCR of a nasopharyngeal swab) and he was told to discontinue the prednisone. COVID-19 testing was positive and he again developed an acute further decline in hearing. He indicated he felt too sick to even perform a home hearing test using the Mimi app. Three weeks later, he required surgical thrombolysis for a deep vein thrombosis of his left leg. He also developed bilateral pulmonary emboli, necessitating treatment with apixaban. On follow up examination four months after the initial sudden SNHL, his hearing had fully recovered (Fig. 1a). Given the incubation time of SARS-CoV-2 infection to disease manifestation, it is likely his sudden SNHL was an initial manifestation of COVID-19.

Patient #2

A 58-year-old white male presented with right-sided hearing loss and tinnitus for two weeks. No associated dyspnea, anosmia, or fever. Physical examination revealed normal appearing ear canals and tympanic membranes. Audiometric testing demonstrated mild to moderate SNHL of the right side (Fig. 1a). He was started on a 60 mg prednisone burst for 4 days, with taper (40 mg PO for 4 days, 20 mg PO for 2 days, 10 mg PO for 2 days). COVID-19 testing was ordered (based on qRT-PCR of a nasopharyngeal swab) and came back positive. Subsequent MRI demonstrated mild maxillary and ethmoid thickening, and normal 7th and 8th nerve complexes. During a one month, telemedicine follow up, the patient reported no change in his unilateral hearing loss and tinnitus, and no additional symptoms. Three months after hearing loss onset, audiometric testing showed slightly worse hearing loss in the low and mid frequencies with recovery in the high frequencies (Fig. 1a). The patient opted against receiving an intratympanic dexamethasone injection.

Patient #3

A 44-year-old Latin-American man with previous history of alcohol-induced dizziness presented with severe vertigo. No associated fever, anosmia, or sore throat. His vertigo was associated with fluctuating sense of imbalance and nausea which coincided with recent airplane travel over a 3-week period. At a 7-day follow up, the patient describes worsening nausea and dizziness, and the onset of tinnitus and hearing loss in the left ear. Audiometric testing revealed bilateral asymmetric sensorineural hearing loss, worse on the left side (Fig. 1a). He was found to have severe gait instability and right beating nystagmus. No facial weakness. He started with vestibular physical therapy and began on a 60mg prednisone burst with anticipated taper after 10 days. Subsequent MRI and MRA did not reveal any cause for the audiovestibular symptoms.

Two-weeks later he began experiencing myalgias, anosmia, cough, sore throat, fever, and ultimately tested positive for COVID-19 (based on qRT-PCR of a nasopharyngeal swab). During a telemedicine visit three days after COVID diagnosis, he complained of left ear pain without an obvious change in hearing. There were no skin lesions or facial weakness. He was started on oral cefpodoxime. During a follow up visit 4 months later he reported his balance was almost fully restored and he did not have vertigo. Ongoing left-sided, high-pitched nonpulsatile tinnitus has persisted and he noted no noticeable difference in hearing between the ears. Objectively, there was mild improvement in the left ear from the initial to the 4 month follow up audiogram with persistent high frequency hearing loss, worse in the left ear (Fig. 1a).

Patient #4

A 68-year-old black female with no previous medical history other than fibrocystic breast disease presented with sore throat, dyspnea, cough, and fever. She had no associated hearing loss or anosmia. COVID-19 testing was positive (based on qRT-PCR of a nasopharyngeal swab). She was started on doxycycline and albuterol before returning to the emergency department 3 days later with worsening dyspnea, cough, and fever. She was admitted for supportive treatment and intubated after 5 days due to progressive tachypnea and hypoxia. She was extubated after 12 days and remained in the hospital for additional supportive care for 2 more weeks until discharged. She did not receive any ototoxic medications while hospitalized. She was first evaluated for sudden hearing loss and right buzzing tinnitus almost 2 months after being taken off the ventilator. Physical examination revealed normal appearing ear canals and tympanic membranes. Audiometric testing showed profound right-sided SNHL (Fig. 1a). She returned 7 days later with sustained vertigo that lasted approximately 10-14 days.

Subsequent MRI of the posterior fossa excluded any retrocochlear pathology to account for her hearing loss and vertigo. During a follow up examination, around 3 and half months after initial sudden SNHL, her hearing showed no improvement (Fig. 1a). Hearing in the contralateral ear also showed no change or decline. A few weeks after follow-up, quantitative IgG testing resulted in a positive antibody concentration.

Patient #5

A 31 year-old white previously healthy Talmud student with no prior otologic history and no active medical problems presented with a sudden right-sided hearing loss. He and his family (wife and two children) were infected with COVID-19 as were many in their Orthodox Jewish community. For the first 10 days before the sudden hearing loss, he experienced what he described as “extreme weakness and fatigue.” He was febrile with a temperature of 101°F (38.3°C). He was symptom-free for one day and then he developed a fever of 102°F (38.9°C) and recurrence of his fatigue and weakness. Two days later he experienced right-sided hearing loss, tinnitus, gravitational receptor (otolithic) dysfunction type of vertigo, ageusia and anosmia. He also experienced chest tightness, but no dyspnea. He did not experience true rotational vertigo. While inpatient COVID-19 testing was available at the time of his infection, outpatient COVID-19 testing was not yet available. Physical examination revealed normal appearing ear canals and tympanic membranes with aerated middle ears bilaterally. His audiogram demonstrated normal left ear hearing and profound right sensorineural hearing loss. He was started on 40 mg prednisone, tapering by 10 mg every three days. Subsequent MRI of the internal auditory canals revealed no retrocochlear pathology to explain his hearing loss. Ten days after starting on prednisone, he received intratympanic dexamethasone (24 mg/mL). There was no response to the oral and intratympanic corticosteroids (Fig. 1a). One month after the onset of his symptoms, COVID-19 testing, based on qRT-PCR of a nasopharyngeal swab, was

negative. Two months after the onset of his symptoms COVID-19 quantitative IgG testing showed a concentration of 85.1 AU/mL (normal range 0-15.0 AU/mL). There was no improvement of his profound hearing loss or tinnitus and no improvement of his aguesia and anosmia. He perceived no chronic vestibular dysfunction.

Patient #6

A 22 year-old previously healthy white woman with no prior otologic history and no active medical problems presented with a sudden right-sided hearing loss and rotational vertigo. She and her family (both parents and three siblings) were infected with COVID-19 as were many in their Orthodox Jewish community. For the first 6 days before the sudden hearing loss, tinnitus and vertigo, she experienced what she described as “extreme fatigue and headache.” She was febrile with a temperature of 103°F (39.4°C). On the seventh day of her illness she experienced right-sided hearing loss, tinnitus, and rotational vertigo. She also experienced mild dyspnea and coughing. The rotational vertigo was unrelenting and lasted two days. Aside from her hearing loss and tinnitus, her symptoms resolved on day 12. Physical examination revealed normal appearing ear canals and tympanic membranes with aerated middle ears bilaterally. She was started on 40 mg prednisone, tapering by 10 mg every three days. Intratympanic dexamethasone injection was offered, but she declined. Her audiometry revealed a right-sided profound hearing loss with no measurable speech discrimination ability (Fig. 1a). While inpatient COVID-19 testing was available at the time of her infection, outpatient COVID-19 testing was not yet available. Subsequent MRI of the internal auditory canals revealed no retrocochlear pathology to explain her sudden hearing loss, tinnitus and vertigo. There was no response to the oral corticosteroids. Nine weeks after the onset of her symptoms, COVID-19 testing, based on qRT-PCR of a nasopharyngeal swab, was negative and COVID-19 quantitative IgG testing

showed a concentration of 93.4 AU/mL (normal range 0-15.0 AU/mL). There was no improvement of her hearing loss or tinnitus. She perceived no chronic vestibular dysfunction.

Patient #7

A 27 year-old previously healthy white man living in New York City with no prior otologic history and no active medical problems presented with a history of recent sudden left-sided hearing loss and rotational vertigo. For the first 6 days before the sudden hearing loss, tinnitus and vertigo, he experienced what he described as “extreme fatigue with intense headache.” He had no ageusia or anosmia. He was febrile with a temperature of 102°F (38.9°C) and experienced repeated shaking and chills. COVID-19 testing, using a qRT-PCR of a nasopharyngeal swab sample, was positive. On the seventh day of his illness he experienced worsening left-sided hearing loss, tinnitus, and rotational vertigo. He had no facial nerve dysfunction. He also experienced mild dyspnea and coughing. The rotational vertigo was unrelenting and lasted two days. Physical examination revealed normal appearing ear canals and tympanic membranes with aerated middle ears bilaterally. Subsequent MRI of the internal auditory canals revealed diffuse contrast uptake and enhancement of the left vestibule, cochlear and vestibular nerves, geniculate ganglion and facial nerve (Fig. 1b). He was started on 60 mg prednisone, tapering by 10 mg every three days. Intratympanic dexamethasone injection was offered, but he declined. Aside from his hearing loss and tinnitus (Fig. 1a), his symptoms resolved on day 12. Initially, there was no response to the oral corticosteroids. At 4 months follow-up his audiogram showed recovery of his hearing loss (Fig. 1a) and he perceived no vestibular dysfunction.

Patient #8

A 72 year-old previously healthy retired white woman presented with bilateral sudden hearing loss and tinnitus. She had several medical problems including essential hypertension, glaucoma and gastroesophageal reflux. For the first 5 days before the sudden hearing loss and tinnitus, she experienced what she described as “extreme fatigue.” She was afebrile throughout her COVID-19 infection. On the fifth day of her illness she experienced bilateral sudden hearing loss and tinnitus. She had no dyspnea, coughing, headache, gastroesophageal disturbance, ageusia or anosmia. Aside from her hearing loss and tinnitus, her fatigue resolved on day 12. Physical examination revealed normal appearing ear canals and tympanic membranes with aerated middle ears bilaterally. She was started on azithromycin 500 mg initially then 250 mg each day for 4 days, and 60 mg prednisone for 7 days, then tapering by 10 mg every day. Intratympanic dexamethasone injection was offered, but she declined. Her initial audiometry revealed bilateral downsloping severe to profound hearing loss with impaired speech discrimination ability (Fig. 1a). Subsequent MRI of the internal auditory canals revealed no retrocochlear pathology to explain her bilateral sudden hearing loss and tinnitus. There was no response to the oral corticosteroids and azithromycin. One month after the onset of her symptoms, COVID-19 quantitative IgG testing was positive. At 4 months after her infection there was mild improvement of her auditory thresholds and no change in her speech discrimination ability (Fig. 1a).

Patient #9

A 46 year-old previously healthy Asian Indian woman with no prior otologic history and no active medical problems presented with a sudden left-sided high frequency hearing loss and tinnitus. Her parents both contracted COVID-19, confirmed by PCR testing. She moved into her parent’s

home to care for them and 7 days later developed cough and influenza-like upper respiratory tract infection symptoms consistent with COVID-19 infection. She had no anosmia or dysgeusia. Because her parents had PCR confirmed COVID-19 infection, she did not leave their home while ill for COVID-19 testing. On day 7 of her infection she developed left-sided sudden hearing loss and tinnitus. She also felt left aural pressure that was not relieved by performing the Valsalva maneuver. Her audiogram (Fig. 1a) showed a high frequency sensorineural hearing loss. She was started on oral prednisone 50 mg daily for 3 days, followed by 10 mg taper every 3 days. She was offered intratympanic dexamethasone, but declined this intervention. There was no response to the oral glucocorticoid. Magnetic resonance imaging of the internal auditory canals revealed no retrocochlear pathology to explain her sudden hearing loss and tinnitus. Nasopharyngeal swab SARS-CoV-2 PCR testing was negative 14 days after her initial symptoms. Her SARS-CoV-2 IgG antibody testing was positive two months after her initial COVID-19 symptoms. Audiometric testing two months after her initial testing showed no improvement or worsening of her hearing (Fig. 1a).

Patient #10

A 61 year-old previously healthy white man with no prior otologic history and no active medical problems presented with right aural pressure, increased tinnitus and episodic true rotational vertigo. His father-in-law contracted COVID-19 while living in a nursing home. His family decided to take him into their home to care for him. Three days after his father-in-law passed away, the patient, his wife and two adult children all became ill. Initially, the patient experienced extreme fatigue and a sense of heaviness. He was mildly febrile with a temperature of 100°F (37.8°C). COVID-19 testing, using a qRT-PCR of a nasopharyngeal swab sample, was positive. By the fifth day he began experiencing labored breathing and a sensation in his chest that lasted two weeks. He had no anosmia or dysgeusia. Three weeks after the onset of his COVID-

19 symptoms he also felt right aural pressure that was not relieved by performing the Valsalva maneuver. He did not perceive a change of his hearing, but his right tinnitus amplitude increased and became bothersome to him. He then had his first episode of true rotational vertigo, nausea and vomiting that lasted 3 hours. His audiogram (Fig. 1a) showed bilateral, right greater than left, down-sloping sensorineural hearing loss. Comparison to a pre-COVID-19 audiogram revealed that he had additional bilateral sensorineural hearing loss. The right aural pressure, tinnitus and episodic true rotational vertigo persisted. He had mild reduction of these symptoms with lowering his daily sodium intake to 1,500-2,000 mg. He was started on oral prednisone 60 mg each day for 3 days and tapering the dose by 10 mg every 3 days. There was a positive response to the oral corticosteroids. During the first 2 weeks of this 18 day course, he noted reduction of his aural pressure and tinnitus with decreased frequency and intensity of his episodic true rotational vertigo. However, as his corticosteroid dose declined his aural pressure and tinnitus increased again. Subsequent magnetic resonance imaging (MRI) of the internal auditory canals revealed no retrocochlear pathology. Audiometric testing 3 months after his initial testing showed mild improvement of his hearing (Fig. 1a). His vestibular function tests showed no vestibular asymmetries in otolithic function or rotational receptor function. Over time, he has experienced continued reduction of his aural pressure and tinnitus and complete resolution of his episodic true rotational vertigo.

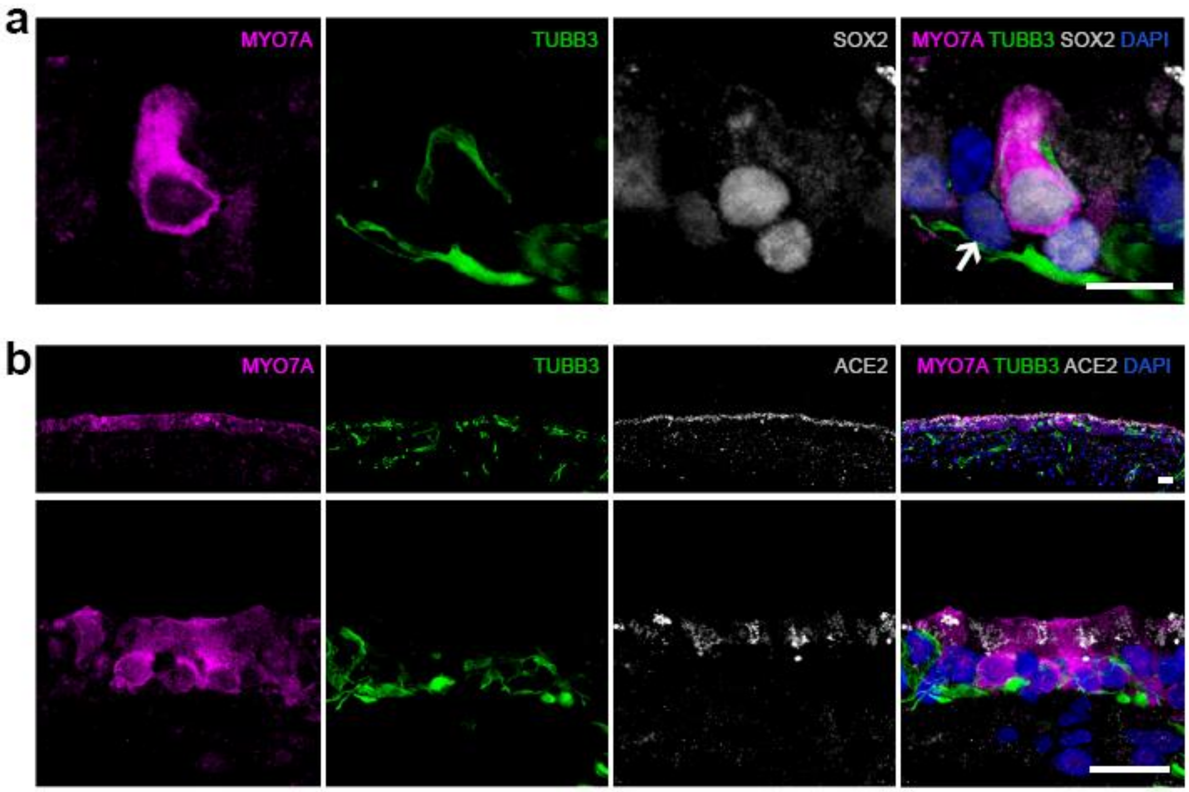

Supplementary Fig. 1 | Co-expression of human vestibular tissue markers and SARS-CoV-2 cell entry-related receptor ACE2. a, The vestibular tissue contains MYO7A+/SOX2+ hair cells and TUBB3+ vestibular ganglion neurites. Arrows indicate SOX2+/MYO7A- supporting cells. Scale bars = 10μm. **b,** IHC images show protein expression of MYO7A (magenta), TUBB3 (green), and ACE2 (gray). The upper images are overview of lower images at higher magnification. Scale bars = 20μm. Representative images from 2 independent experiments with similar results.

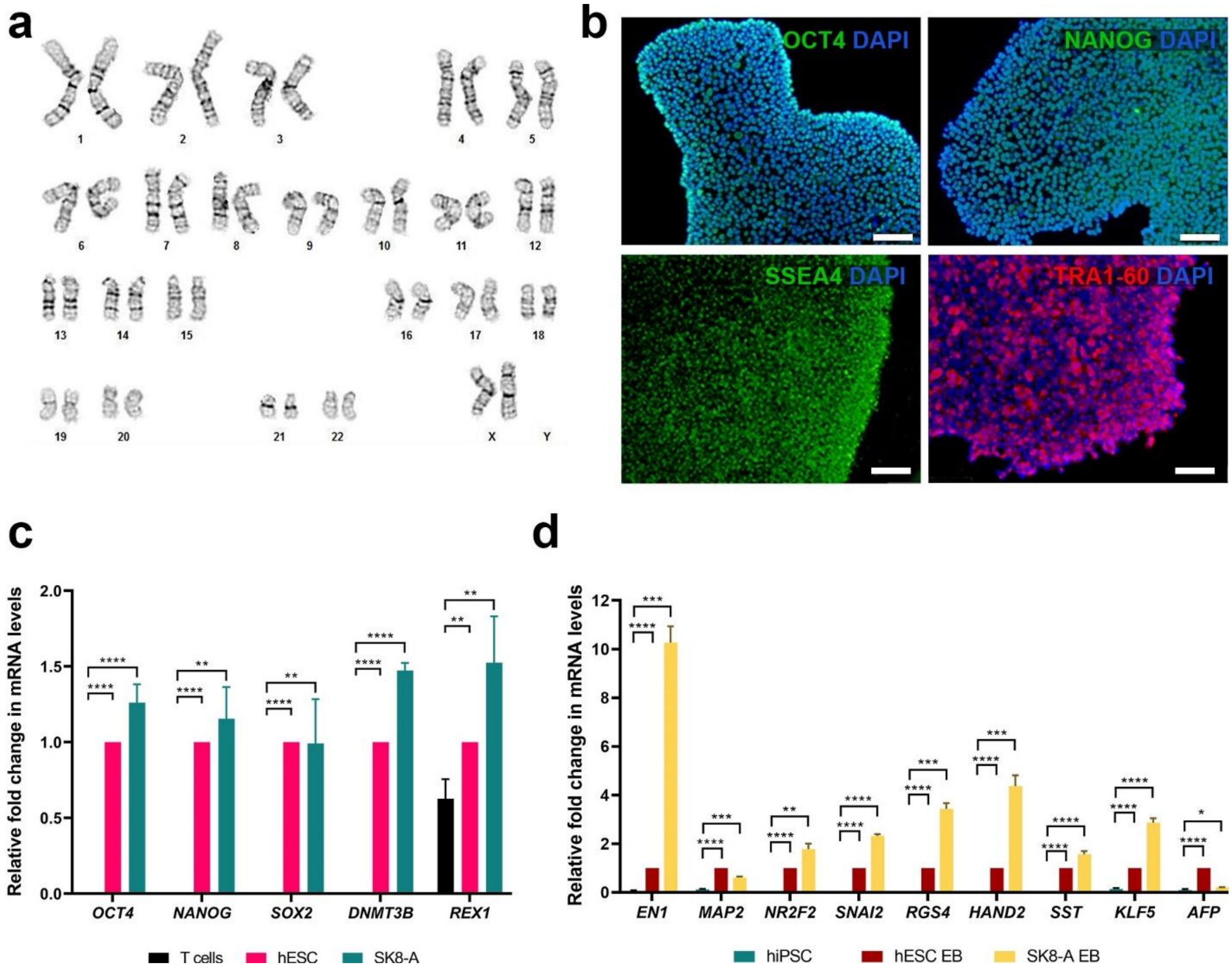

Supplementary Fig. 2 | Characterization of hiPSC-line SK8-A. a, Chromosome analysis shows a normal karyotype. No detection of clonal abnormalities at the stated band level of resolution. **b**, Immunofluorescence staining shows presence of pluripotency markers OCT4, NANOG, SSEA4 and TRA-1-60. Scale bars = 100µm. **c**, Bar graphs with qRT-PCR data show the relative expression levels of pluripotency markers. T cells and hESC are used as negative and positive control, respectively. **d**, qRT-PCR data from EBs shows hiPSC-line SK8-A has a functional ability of differentiation into trilineage. *EN1*, *MAP2* and *NR2F2* are ectoderm makers, *SNAIL2*, *RGS4* and *HAND2* are mesoderm markers and *SST*, *KLF5* and *APF* are endoderm markers. n = 1 biological replicate, 3 technical replicates; *P<0.05, **P<0.01, ***P<0.001,

****P<0.0001; mean ± SEM. Abbreviations: hESC, human embryonic stem cells; SSEA4, stage-specific embryonic antigen-4; DNMT3, DNA Methyltransferase 3 Beta; hTERT, Telomerase reverse transcriptase; EN1, Engrailed Homeobox 1; MAP1, Microtubule Associated Protein 2; NR2F2, Nuclear Receptor Subfamily 2 Group F Member 2; RGS4, Regulator Of G Protein Signaling 4; HAND2, Heart- and neural crest derivatives-expressed protein 2; SST, Somatostatin; KLF5, Kruppel Like Factor 5; APF, Aggregation-promoting factor; EB, embryoid body.

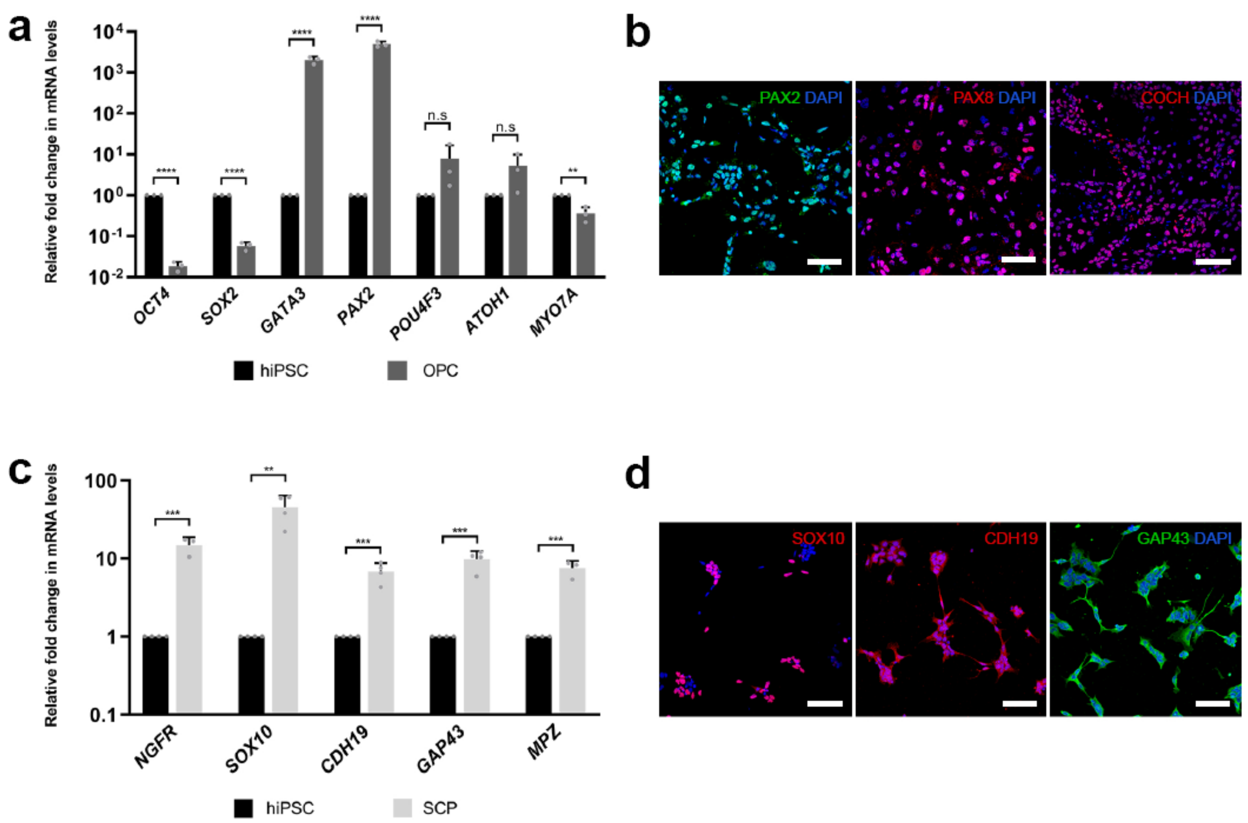

Supplementary Fig. 3 | Characterization of OPCs and SCPs generated from hiPSC line UCSD112i-2-11. a, qRT-PCR assays of transcripts for otic markers in OPCs on day 20, plotted relative to the expression in hiPSCs, controlled for the amount of isolated RNA. Individual gene expression levels in hiPSCs were set to 1. **b**, immunocytochemistry assay for early otic lineage markers in OPCs. Scale bars = 100μm. **c**, mRNA expression of Schwann cell lineage markers in SCPs, plotted relative to the expression in hiPSCs, controlled for the amount of isolated RNA. Individual gene expression levels in hiPSCs are set to 1. **d**, Protein expression of Schwann cell precursor markers in SCPs. Scale bars = 100μm. n = 3 biological replicates, 3 technical replicates; n.s>0.05, **P<0.01, ***P<0.001, ****P<0.0001; mean ± SEM.

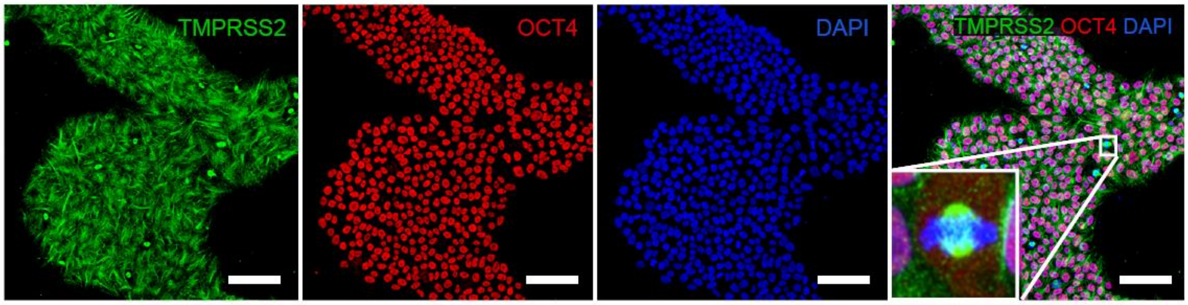

Supplementary Fig. 4 | High expression of SARS-CoV-2 cell entry-related proteases TMPRSS2 in hiPSCs. During mitotic phase of hiPSC, TMPRSS2 is involved in microtubules to move chromosome apart which indicated by the rectangle and this is shown at higher magnification. Representative images from at least 3 independent experiments with similar results. Scale bars = 100μm.

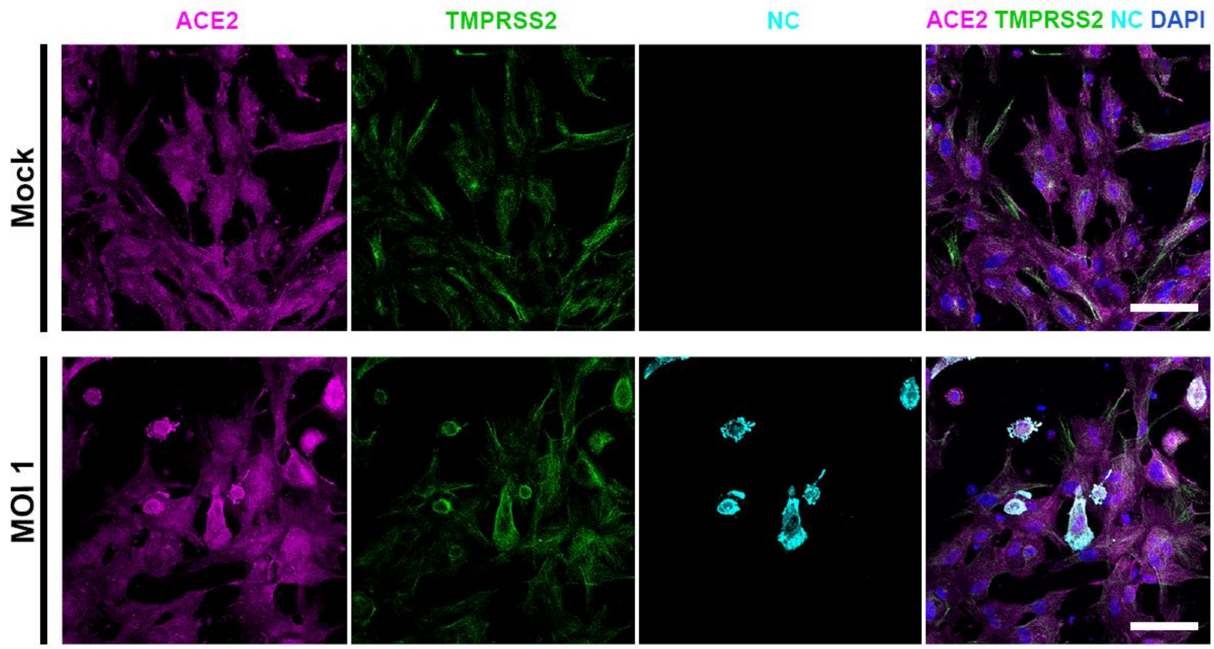

Supplementary Fig. 5 | SARS-CoV-2 infects OPCs differentiated from hiPSC line SK8-A. Infected OPCs are visualized by immunostaining for viral NP and co-immunostaining for ACE2 and TMPRSS2 at 48 hpi. Mock-infected negative control OPCs are compared to SARS-CoV-2 infection at MOI = 1. Data are representative images from 2 independent infections. Scale bars = 100μm.

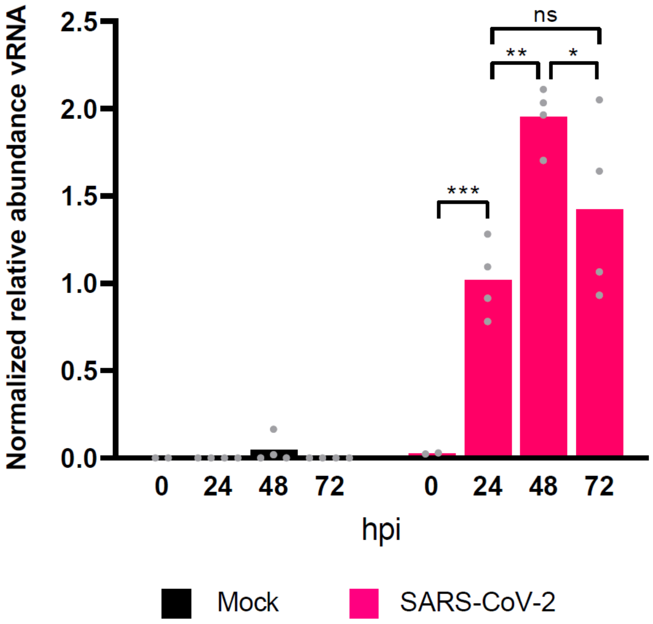

Supplementary Fig. 6 | Detection of SARS-CoV-2 RNA in supernatant of OPCs. It was normalized to presence of cell-free 18s rRNA in supernatant. Except for zero hour sample, samples harvested at each time point represent viral RNA released over previous 24 hours. 24 hpi was used as the reference control group. Bar height represents geometric mean between two experiments, each with two nested replicates. Points represent individual samples. P-values were determined by F-test (comparison of ANOVA models) (*** $0<p<0.001$; ** $0.001<p<0.01$; * $0.01<p<0.05$; ns non-significant).

Supplementary Table 1: Primers used for qRT-PCR.

Gene	Primer forward (5' → 3')	Primer reverse (5' → 3')	Product size (bp)
ACE2	TGCAGACCAAAGCATCAAAG	AATTAGCCACTCGCACATCC	185
TMPRSS2	CACTGTGCATCACCTTGACC	ACACACCGATTCTCGTCCTC	196
FURIN	ACAACTATGGGACGCTGACC	TGGACACAGCTCTTCTGGTG	163
OCT4	CCTGAAGCAGAAGAGGATCACC	AAAGCGGCAGATGGTCGTTTGG	106
SOX2	GCTACAGCATGATGCAGGACCA	TCTGCGAGCTGGTCATGGAGTT	135
GATA3	ACCACAACCACACTCTGGAGGA	TCGGTTTCTGGTCTGGATGCCT	132
PAX2	GACTATGTTCGCCTGGGAGATTC	AAGGCTGCTGAACTTTGGTCCG	119
POU4F3	CAGTCTGCACTCTGGCTCCGA	GTTCTTGCCGTGGGAGACGATA	148
ATOH1	CCTTCCAGCAAACAGGTGAATGG	GAACGACGGGATAACATTGCGC	126
MYO7A	CTCAAACAGCCACTGCTCTACC	CTCATGGCTGTGTGGTACTTGG	122
NGFR	TGGCCTACATAGCCTTCAAGA	GAGATGCCACTGTCGCTGT	124
SOX10	CCTCACAGATCGCCTACACC	CATATAGGAGAAGGCCGAGTAGA	161
CDH19	ACAAGCGTCTGTAACTCTGGG	AGCAAACTTCGTGTTGGACA	116
GAP43	GGCCGCAACCAAAATTCAGG	CGGCAGTAGTGGTGCCTTC	167
MPZ	AAGTGCCAACTAGGTACGGG	CATAGCACTGAGCCTCCTCT	146
GAPDH	ACAACTTTGGTATCGTGGAAGG	GCCATCACGCCACAGTTTC	101
NANOG	TCCAACATCCTGAACCTCAG	GACTGGATGTTCTGGGTCTG	260
DNMT3B	ATAAGTCGAAGGTGCGTCGT	GGCAACATCTGAAGCCATTT	203
REX1	TGGACACGTCTGTGCTCTTC	GTCTTGGCGTCTTCTCGAAC	181
β-ACTIN	GGACTTCGAGCAAGAGATGG	AGCACTGTGTTGGCGTACAG	234
2019-nCoV_N1	GACCCCAAAATCAGCGAAAT	TCTGGTTACTGCCAGTTGAATCTG	
EN1	Thermofisher, Assay ID# Hs00154977_m1		
MAP2	Thermofisher, Assay ID# Hs00154977_m1		
NR2F2	Thermofisher, Assay ID# Hs00819630_m1		
SNALI2	Thermofisher, Assay ID# Hs00161904_m1		
RGS4	Thermofisher, Assay ID# Hs01111690_g1		
HAND2	Thermofisher, Assay ID# Hs00232769_m1		
SST	Thermofisher, Assay ID# Hs00356144_m1		
KLF5	Thermofisher, Assay ID# Hs00156145_m1		
AFP	Thermofisher, Assay ID# Hs01040598_m1		
2019-nCoV_N1-	FAM-ACC CCG CAT TAC GTT TGG TGG ACC-BHQ1		

Supplementary Table 2: Primary antibodies used for immunostaining.

Antigen	Manufacturer	Host and Clonality	Dilution
ACE2	Novus Biologicals NBP1-76614	Rabbit polyclonal	1:100 - 600
ACE2	R&D Systems AF933	Goat polyclonal	3ug/ml
TMPRSS2	Millipore Sigma MABF2158	Mouse monoclonal IgG1	1:100
FURIN	Novus Biologicals NB100-1903	Rabbit polyclonal	1:250
FURIN	Enzo Life Sciences ALX-803-015-	Mouse monoclonal IgG2a	1:250
SARS-CoV-2 Nucleocapsid	GeneTex GTX135357	Rabbit polyclonal	1:500
J2	Scicons 10010200	Mouse monoclonal IgG2a	1:2000
MAP2	EnCor Biotechnology CPCA-MAP2	Chicken polyclonal	1:10,000
SSEA4	DSHB MC-813-70	Mouse monoclonal IgG3	2 μg/ml
OCT4	Cell Signaling Technology 2750S	Rabbit polyclonal	1:200
NANOG	Abcam ab173368	Mouse monoclonal IgG	1:100
PAX2	R&D Systems AF3364	Goat polyclonal	1:100
PAX8	Abcam ab97477	Rabbit polyclonal	1:200
COCH	Millipore MABF267	Rat monoclonal	1:100
MYO7A	Proteus BioSciences	Rabbit polyclonal	1:500
SOX2	BD 561469	Mouse monoclonal IgG1	1:200
TUBB3	BioLegend	Mouse monoclonal IgG2a	1:1000
GAP43	Novus Biologicals NBP1-41123	Sheep polyclonal	1:250
SOX10	Cell Signaling Technology 89356	Rabbit monoclonal	1:500
CDH19	Abnova H00028513-A01	Mouse polyclonal	1:250
OCT4	Abcam ab19857	Rabbit polyclonal	1:200
NANOG	Abcam ab21624	Rabbit polyclonal	1:50
TRA1-60	Millipore MAB4360	Mouse monoclonal IgM	1:200
SSEA4	Millipore MAB4304	Mouse monoclonal IgG3	1:200

Supplementary Table 3: Secondary antibodies used for immunostaining.

Host and Species Reactivity	Manufacturer	Conjugate	Dilution
Goat anti mouse IgG	Thermofisher A11001	Alexa fluor 488	1:2000
Donkey anti rabbit	Thermofisher A10042	Alexa fluor 568	1:500
Goat anti rabbit	Thermofisher A11008	Alexa fluor 488	1:1000
Goat anti mouse IgG1	Thermofisher A21124	Alexa fluor 568	1:500
Goat anti mouse IgG2a	Thermofisher A21241	Alexa fluor 647	1:400
Chicken anti goat	Thermofisher A21467	Alexa fluor 488	1:500
Goat anti rat	Thermofisher A11007	Alexa fluor 594	1:500
Donkey anti rabbit	Thermofisher A31573	Alexa fluor 647	1:1000
Chicken anti goat	Thermofisher A21469	Alexa fluor 647	1:1000
Donkey anti mouse	Abcam ab150110	Alexa fluor 555	1:1000
Donkey anti goat	Thermofisher A32814	Alexa fluor 488	1:1000
Donkey anti rabbit	Abcam ab150075	Alexa fluor 647	1:1000
Goat anti mouse	Thermofisher A28175	Alexa fluor 488	1:1000
Goat anti Mouse IgG2a	Thermofisher A21131	Alexa fluor 488	1:2000
Donkey anti Mouse IgG	Thermofisher A31571	Alexa fluor 647	1:1000
Goat anti Mouse IgM	Thermofisher A21426	Alexa fluor 555	1:1000
Goat anti Chicken IgY	Thermofisher A11039	Alexa fluor 488	1:1000
