# Supplementary material for: Direct SARS-CoV-2 infection of the human inner ear may underlie COVID-19-associated audiovestibular dysfunction
Source: Commun Med (Lond). 2021 Oct 29;1:44. doi: 10.1038/s43856-021-00044-w (PMC8633908; doi:10.1038/s43856-021-00044-w)
Supplement: Supplementary file 4 — Description of Additional Supplementary Files. [file 43856_2021_44_MOESM4_ESM.pdf]

Description of Additional Supplementary Files

File Name: Supplementary Data 1

Description: Clinical Characteristics of Study Patients.

[a]R, right-sided; L, left-sided; B, bilateral; SNHL, sensorineural hearing loss; HL, hearing loss
[b]+, symptoms present
[c]-, symptoms absent
[d]Hearing loss and recovery are referenced from Fig. 1a.

File Name: Supplementary Data 2

Description: The source data for all main figures.
