# Supplementary material for: Direct SARS-CoV-2 infection of the human inner ear may underlie COVID-19-associated audiovestibular dysfunction
Source: Commun Med (Lond). 2021 Oct 29;1:44. doi: 10.1038/s43856-021-00044-w (PMC8633908; doi:10.1038/s43856-021-00044-w)
Supplement: Supplementary file 6 — Reporting summary. [file 43856_2021_44_MOESM6_ESM.pdf]

nature research

Corresponding author(s): Konstantina Stankovic

Last updated by author(s): Jun 10, 2021

Reporting Summary

Nature Research wishes to improve the reproducibility of the work that we publish. This form provides structure for consistency and transparency in reporting. For further information on Nature Research policies, see our Editorial Policies and the Editorial Policy Checklist.

Statistics

For all statistical analyses, confirm that the following items are present in the figure legend, table legend, main text, or Methods section.

n/a	Confirmed	
☐	☒	The exact sample size (n) for each experimental group/condition, given as a discrete number and unit of measurement
☐	☒	A statement on whether measurements were taken from distinct samples or whether the same sample was measured repeatedly
☐	☒	The statistical test(s) used AND whether they are one- or two-sided<br>*Only common tests should be described solely by name; describe more complex techniques in the Methods section.*
☒	☐	A description of all covariates tested
☒	☐	A description of any assumptions or corrections, such as tests of normality and adjustment for multiple comparisons
☐	☒	A full description of the statistical parameters including central tendency (e.g. means) or other basic estimates (e.g. regression coefficient) AND variation (e.g. standard deviation) or associated estimates of uncertainty (e.g. confidence intervals)
☐	☒	For null hypothesis testing, the test statistic (e.g. F, t, r) with confidence intervals, effect sizes, degrees of freedom and P value noted<br>*Give P values as exact values whenever suitable.*
☒	☐	For Bayesian analysis, information on the choice of priors and Markov chain Monte Carlo settings
☒	☐	For hierarchical and complex designs, identification of the appropriate level for tests and full reporting of outcomes
☒	☐	Estimates of effect sizes (e.g. Cohen's d, Pearson's r), indicating how they were calculated

Our web collection on statistics for biologists contains articles on many of the points above.

Software and code

Policy information about availability of computer code

Data collection	n/a
Data analysis	n/a

For manuscripts utilizing custom algorithms or software that are central to the research but not yet described in published literature, software must be made available to editors and reviewers. We strongly encourage code deposition in a community repository (e.g. GitHub). See the Nature Research guidelines for submitting code & software for further information.

Data

Policy information about availability of data

All manuscripts must include a data availability statement. This statement should provide the following information, where applicable:

- Accession codes, unique identifiers, or web links for publicly available datasets
- A list of figures that have associated raw data
- A description of any restrictions on data availability

All data generated or analyzed during this study are included in this published article and its supplementary information files. Source data for the main figures of the manuscript is available as Supplementary Data 2. All other data are available from the corresponding author on reasonable request.

Field-specific reporting

Please select the one below that is the best fit for your research. If you are not sure, read the appropriate sections before making your selection.

☒ Life sciences ☐ Behavioural & social sciences ☐ Ecological, evolutionary & environmental sciences

For a reference copy of the document with all sections, see nature.com/documents/nr-reporting-summary-flat.pdf

Life sciences study design

All studies must disclose on these points even when the disclosure is negative.

Sample size	We did 3 independent experiments with at least 3 technical replicates except for experiments with surgical tissue which is impossible to get more than 2. And some of experiments related to viral infection because these are experiments done in the BSL3 and therefore technically and physically demanding.
Data exclusions	No data exclusions.
Replication	We confirm that attempts at replication were successful.
Randomization	This is not relevant to our study because our main goal is not a comparison between groups.
Blinding	This is not relevant to our study because our main goal is not a comparison between groups.

Reporting for specific materials, systems and methods

We require information from authors about some types of materials, experimental systems and methods used in many studies. Here, indicate whether each material, system or method listed is relevant to your study. If you are not sure if a list item applies to your research, read the appropriate section before selecting a response.

Materials & experimental systems

n/a	Involved in the study	
☐	☒	Antibodies
☐	☒	Eukaryotic cell lines
☒	☐	Palaeontology and archaeology
☐	☒	Animals and other organisms
☐	☒	Human research participants
☒	☐	Clinical data
☒	☐	Dual use research of concern

Methods

n/a	Involved in the study	
☒	☐	ChIP-seq
☒	☐	Flow cytometry
☒	☐	MRI-based neuroimaging

Antibodies

Antibodies used

ACE2: Novus Biologicals NBP1-76614, Polyclonal, LOT 3215-2001
ACE2: R&D Systems AF933, Polyclonal, LOT HOK0320051
TMPRSS2: Millipore Sigma MABF2158, clone P5H9-A3, LOT 3474105
FURIN: Novus Biologicals NB100-1903, Polyclonal, lot VE300810
FURIN: ALX-803-015-R100, MON-148, Monoclonal, LOT 09111725
SARS-CoV-2: GeneTex GTX135357, Polyclonal,
J2: Scicons 10010200
MAP2: EnCor Biotechnology CPCA-MAP2, Polyclonal
SSEA4: DSHB MC-813-70, Monoclonal
OCT4: Cell Signaling Technology 2750S, Polyclonal, LOT 4
NANOG: Abcam ab173368, Mouse monoclonal [23D2-3C6],
PAX2: R&D Systems AF3364, Polyclonal, LOT XOT0319101
PAX8: Abcam ab97477, Polyclonal, LOT GR312954-17
COCH: Millipore MABF267, 9A10D2, LOT 3068048
MYO7A: Proteus BioSciences 25-6790, Polyclonal, LOT 110119
SOX2: BD 561469, Mouse monoclonal
TUBB3: BioLegend 801203, Mouse monoclonal, LOT B300435
GAP43: Novus Biologicals NBP1-41123, Polyclonal, LOT B-2
SOX10: Cell Signaling Technology 89356, Polyclonal, LOT 1
CDH19: Abnova H00028513-A01, Polyclonal, LOT 11109
OCT4: Abcam ab19857, Polyclonal
NANOG: Abcam ab21624, Polyclonal
TRA1-60: Millipore MAB4360, Mouse monoclonal

	SSEA4: Millipore MAB4304, clone MC-813-70
Validation	ACE2: Novus Biologicals NBP1-76614, https://www.novusbio.com/products/ace-2-antibody_nbp1-76614, ACE2: R&D Systems AF933, https://www.rndsystems.com/products/human-mouse-rat-hamster-ace-2-antibody_af933<br>TMPRSS2: Millipore Sigma MABF2158, https://www.emdmillipore.com/US/en/product/Anti-TMPRSS2-Antibody-clone-P5H9-A3,MM_NF-MABF2158-100UG<br>FURIN: Novus Biologicals NB100-1903, https://www.novusbio.com/products/furin-antibody_nb100-1903<br>FURIN: ALX-803-015-R100, https://www.enzolifesciences.com/ALX-803-015/furin-convertase-r2-monoclonal-antibody-mon-148/<br>SARS-CoV-2: GeneTex GTX135357, https://www.genetex.com/Product/Detail/SARS-CoV-2-COVID-19-nucleocapsid-antibody/GTX135357<br>J2: Scicons 10010200, https://scicons.eu/en/antibodies/j2/<br>MAP2: https://encorbio.com/product/cpca-map2ab/<br>SSEA4: DSHB MC-813-70, https://dshb.biology.uiowa.edu/MC-813-70-SSEA-4<br>OCT4: Cell Signaling Technology 2750S, https://www.cellsignal.com/products/primary-antibodies/oct-4-antibody/2750<br>NANOG: Abcam ab173368, https://www.abcam.com/nanog-antibody-23d2-3c6-ab173368.html<br>PAX2: R&D Systems AF3364, https://www.rndsystems.com/products/human-pax2-antibody_af3364<br>PAX8: Abcam ab97477, https://www.abcam.com/pax8-antibody-ab97477.html<br>COCH: Millipore MABF267, https://www.emdmillipore.com/US/en/product/Anti-Cochlin-Antibody-COCH-clone-9A10D2,MM_NF-MABF267<br>MYO7A: Proteus BioSciences 25-6790, http://www.proteus-biosciences.com/Product/View/Myosin-VIIa-248.aspx<br>SOX2: BD 561469, https://www.bdbiosciences.com/us/applications/research/intracellular-flow/intracellular-antibodies-and-isotype-controls/anti-human-antibodies/purified-mouse-anti-sox2-o30-678/p/561469<br>TUBB3: BioLegend 801203, https://www.biolegend.com/de-de/products/alexa-fluor-488-anti-tubulin-beta-3-tubb3-antibody-10828<br>GAP43: Novus Biologicals NBP1-41123, https://www.novusbio.com/products/gap-43-antibody_nbp1-41123<br>SOX10: Cell Signaling Technology 89356, https://www.cellsignal.com/products/primary-antibodies/sox10-d5v9l-rabbit-mab/89356<br>CDH19: Abnova H00028513-A01, http://www.abnova.com/products/products_detail.asp?catalog_id=H00028513-A01<br>OCT4: Abcam ab19857, https://www.abcam.com/oct4-antibody-ab19857.html<br>NANOG: Abcam ab21624, https://www.abcam.com/nanog-antibody-ab21624.html<br>TRA1-60: Millipore MAB4360, https://www.emdmillipore.com/US/en/product/Anti-TRA-1-60-Antibody-clone-TRA-1-60,MM_NF-MAB4360<br>SSEA4: Millipore MAB4304, https://www.emdmillipore.com/US/en/product/Anti-Stage-Specific-Embryonic-Antigen-4-Antibody-clone-MC-813-70,MM_NF-MAB4304

Eukaryotic cell lines

Policy information about cell lines

Cell line source(s)	SK8-A: healthy subject's primary dermal fibroblasts<br>UCSD112i-2-11: Skin Fibroblast
Authentication	*Describe the authentication procedures for each cell line used OR declare that none of the cell lines used were authenticated.*
Mycoplasma contamination	We confirm that all cell lines tested negative for mycoplasma contamination.
Commonly misidentified lines<br>(See ICLAC register)	n/a

Animals and other organisms

Policy information about studies involving animals; ARRIVE guidelines recommended for reporting animal research

Laboratory animals	Wild-type CBA/CaJ mice were obtained from the Jackson Laboratory (Bar Harbor, ME). Animals of either sex were used for experimentation in an estimated 50/50 ratio. 6-week old mice were sacrificed through cardiac perfusion, decapitated and inner ears were removed.
Wild animals	n/a
Field-collected samples	n/a
Ethics oversight	This research was approved by the<br>Institutional Animal Care and Use Committees at Massachusetts Eye and Ear.

Note that full information on the approval of the study protocol must also be provided in the manuscript.

Human research participants

Policy information about studies involving human research participants

Population characteristics	Inclusion criteria was as follows for the retrospective portion: patients with a confirmed COVID-19 PCR test and experiencing any of the audiovestibular symptoms stated in our manuscript. Only adults were asked for access to their medical charts and all studies performed were retrospective as data utilized was per standard of care. Population was not limited to a specific ethnic group or sex as COVID-19 and audiovestibular symptoms are not specific to one said group.

	Inclusion criteria was as follows for collecting labyrinthectomy tissue: adults (ages 18+), not limited to a specific sex or ethnic group, undergoing a labyrinthectomy or a translabyrinthine resection of an acoustic neuroma.
Recruitment	Data utilized for this manuscript were obtained after verbal consent was obtained from patients by their otologic physician (co-authors included here). All data/information were collected per standard of care and studied retrospectively for this paper.<br><br>Use of human labyrinthine tissue was was reviewed by the Massachusetts General Brigham Institutional Review Board and determined to be Exempt, thus consent was not required.
Ethics oversight	The study of patients with COVID-19 associated sudden audiovestibular symptoms was approved by the Massachusetts General Brigham Institutional Review Board (2020P002900). Verbal informed consent was obtained from all subjects and all procedures were conducted in accordance with the Helsinki Declaration. This research was reviewed by the Massachusetts General Brigham Institutional Review Board and determined to be Exempt (2020P003329).

Note that full information on the approval of the study protocol must also be provided in the manuscript.
